# Cytological and biophysical comparative analysis of cell structures at the microsporogenesis stage in sterile and fertile *Allium* species

**DOI:** 10.1007/s00425-016-2597-0

**Published:** 2016-09-29

**Authors:** Dorota Tchórzewska, Kamil Deryło, Krystyna Winiarczyk

**Affiliations:** 1 Department of Plant Anatomy and Cytology, Maria Curie-Skłodowska University, Akademicka 19 Street, 20-033 Lublin, Poland; 2 Department of Molecular Biology, Maria Curie-Skłodowska University, Lublin, Poland

**Keywords:** *Allium sativum*, *A. ampeloprasum*, Male sterility, Callose wall, Sporoderm, Autofluorescence-spectral imaging

## Abstract

**Using a live-cell-imaging approach and autofluorescence-spectral imaging, we showed quantitative/qualitative fluctuations of chemical compounds within the meiocyte callose wall, providing insight into the molecular basis of male sterility in plants from the genus** ***Allium***.

*Allium sativum* (garlic) is one of the plant species exhibiting male sterility, and the molecular background of this phenomenon has never been thoroughly described. This study presents comparative analyses of meiotically dividing cells, which revealed inhibition at the different microsporogenesis stages in male-sterile *A. sativum* plants (cultivars Harnas and Arkus) and sterile *A. ampeloprasum* var. *ampeloprasum* (GHG-L), which is phylogenetically related to garlic. Fertile species *A. ampeloprasum* (leek) was used as the control material, because leek is closely related to both garlic and GHG-L. To shed more light on the molecular basis of these disturbances, autofluorescence-spectral imaging of live cells was used for the assessment of the biophysical/biochemical differences in the callose wall, pollen grain sporoderm, and the tapetum in the sterile species, in comparison with the fertile leek. The use of techniques for live-cell imaging (autofluorescence-spectral imaging) allowed the observation of quantitative/qualitative fluctuations of autofluorescent chemical compounds within the meiocyte callose wall. The biophysical characterisation of the metabolic disturbances in the callose wall provides insight into the molecular basis of male sterility in *A. sativum*. In addition, using this method, it was possible for the first time, to determine precisely (on the basis of fluctuations of autofluorescence compounds) the meiosis stage in which normal microsporogenesis is disturbed, which was not visible using light microscopy.

## Introduction

Through unlimited genetic fluctuations, sexual reproduction contributes to not only dynamic development and spread of organisms, but also their adaptation to environmental changes. Some plants, however, have lost the capability of this type of reproduction. This phenomenon is referred to as sterility, which involves the inability to produce functional gametes and viable zygotes emerging in the fertilisation process (Kaul 1988). Many processes are involved in the formation of elements associated with sexual reproduction, from mitotic divisions in the primordia through cytological cell differentiation and reduction divisions to the final process of gametogenesis. Although the success of sexual reproduction depends on the co-ordination of the numerous transformations that take place in the entire flower, a special role is ascribed to all processes occurring within anthers and ovaries. Disturbances in the development of both male (anther) and female (pistil) structures lead to the formation of abnormal, non-viable gametophytes, which are the major cause of sterility. A relatively frequent phenomenon in plants is irregularity in the development of male reproductive cells. The most comprehensive classification proposed by Kaul (1988, 2012) distinguishes between genetic (phenotypic and genotypic) and non-genetic (chemical, physiological, and ecological) causes of male sterility. This classification describes the great number of factors that contribute to developmental disturbances and that take place during the formation of male gametophytes capable of fertilisation. Male gametophytes (pollen grains) are formed in the anther in a multi-step process called microsporogenesis, and are developed from pollen mother cells (PMC). These cells are present among sporophytic cells (tapetum) that are involved in, e.g., nutrient and enzyme supply, indispensable at all the stages of cell differentiation (Taylor et al. 1997; Wang et al. 2003; Lerstern 2004). Disturbances in the development and function of the tapetum have been described as a frequent cause of male sterility in plants, e.g., in *Zea mays* (Chaubal et al. 2000), *Allium schoenoprasum* (Engelke et al. 2002), *Beta vulgaris* (Chauhan 2005), *B. campestris* (Xie et al. 2005), and *Arabidopsis* (Ma 2005).

*Allium sativum* (garlic) is one of the plant species exhibiting male sterility. The plant is important for humans and has been used for centuries due to its content of biologically active compounds positively affecting human health. Cultivated on an industrial scale, *A. sativum* is propagated solely in the vegetative way. On the one hand, vegetative propagation prevents genetic variability, and the parent plant is maintained (a desirable feature for industrial plant production); on the other hand, vegetative propagation prevents the genetic variability provided by meiotic division. Sexual reproduction is of course the simplest and the best way of producing new varieties in agriculture, characterised by desirable industrial traits, and also is the only natural method of adaptation to changing environmental conditions.

Since, garlic is cultivated on all continents, and in various climatic conditions, elucidation of the causes of sterility and overcoming it would be highly valuable. It is currently agreed that cultivated garlic has lost the ability to reproduce sexually, and there are several hypotheses about the cause (Kononkov 1953; Koul and Gohil 1970; Novak 1972; Konvicka 1973; Etoh 1986). It has been postulated (Shemesh Mayer et al. 2013), that garlic sterility may be caused by both genetic and environmental factors, and several possible types of sterility have been defined, including: completely sterile type 1 (disturbances in the male and female lines); male sterility type 2 (disturbances only in the male line at the microsporogenesis stage); male sterility type 3 (pollen grains are formed, but they are sterile); and female sterility (Shemesh Mayer et al. 2013). It must be noted that, although there are many manifestations of garlic sterility, there have so far been no comprehensive reports on the mechanisms responsible for these phenomena.

This study presents an analysis of the microsporogenesis process in male-sterile *A. sativum* L. plants (cultivars Harnas and Arkus) and *A. ampeloprasum* var. *ampeloprasum* (commonly called great-headed garlic—GHG), which is phylogenetically related to garlic (Najda et al. 2016). *A. ampeloprasum* L. (leek), a fertile species closely related to both garlic and GHG-L (Tchórzewska et al. 2015), was used as the control material. The comparative analyses of meiotically dividing cells revealed inhibition at the different microsporogenesis stages in the sterile species. To shed more light on the molecular basis of these disturbances, autofluorescence-spectral imaging, a method for imaging live cells and assessing the biophysical/biochemical differences in the analysed plant material, was employed. Particular attention was paid to the biophysical and biochemical parameters of the microsporocyte callose wall, pollen grain sporoderm, and the tapetum in the sterile species, in comparison with the fertile leek. Significant quantitative and qualitative differences were observed in the callose wall and sporoderm, which for the first time allowed identification of subtle changes in the key microsporogenesis structures. This also allowed precise identification of the point of emergence of the disturbances in the normal development of the male gametophyte leading to a complete inhibition of the process. Thus, these investigations significantly expand our knowledge about possible mechanisms which may cause male sterility in commercially important plants from the genus *Allium*.

## Materials and methods

### Plant material

*Allium sativum* (cultivars Harnas and Arkus), *A. ampeloprasum* var. *ampeloprasum* (great-headed garlic cultivar—GHG-L, GenBank numbers: KT809295, and KT809296), and *A. ampeloprasum* (leek) were the research material. All plants were collected from the Botanical Garden of Maria Curie-Skłodowska University in Lublin. *A. sativum* (both cultivars) and GHG-L were propagated exclusively in the vegetative mode using daughter bulbs, the so-called cloves, while *A. ampeloprasum* was propagated from seeds.

Meiocytes used in the analysis of microsporogenesis were sampled from anthers at different developmental stages from closed flower buds and spathe-covered inflorescences. Pollen grains were collected from anthers of flowers at the anthesis stage after the opening of the inflorescence spathe. The material was sampled randomly from 50 plants throughout the microsporogenesis taking place in the anthers (ca. 1 per month). Each microsporogenesis stage was analysed in a minimum of 40 meiotically dividing cells.

### Transmission light microscopy (LM)

The anthers of the investigated species were crushed and stained with acetocarmine (Gerlach 1977). *A. sativum* cv. Arkus and *A. ampeloprasum* pollen grains were collected immediately at the anthesis stage and stained with the Alexander assay according to the method of Peterson et al. (2010). The observations were carried out under a light microscope Nikon Eclipse Ni with Nomarski contrast. Photographic documentation was made with a digital camera and NIS-Elements BP software.

### Confocal microscopy (CM)

To perform the observations with laser scanning confocal microscopy, crushed preparations of the anthers of all the species at different developmental stages were made. The anthers were placed in distilled water and immediately analysed under an LSM780 Zeiss microscopy system build around an AxioObserverZ.1 inverted microscope equipped with a Plan-Apochromat 63×/1.40 Oil DIC M27 objective. For autofluorescence-spectral imaging (ASI), the 32 channel GaAsP spectral detector was used with freely selectable range (resolution down to 3 nm). The microscope was equipped with the whole range of lasers lines (405, 458, 488, 514, 543, and 633 nm). Galleries of spectral images and emission spectra from meiocytes were obtained using a 405 nm laser set at 3 % power, and the GaAsP detector was set for the range 411–692 nm. The pinhole diameter was set to 1AU. A lambda-coded image composed of 32 images was obtained, with each image acquired with a separate narrow bandwidth, representing the complete spectral distribution of the fluorescence signals for every point of the microscopic image. Simultaneously, the images in the T-PMT (transmission light) mode were obtained using the same laser line as a light source. Each experiment was repeated with five samples per different stage, and the most representative cell was used. Post-acquisition image analysis was carried out using the advanced linear unmixing function (Zen2010 software, Zeiss), which separates mixed fluorescence signals pixel by pixel using the entire emission spectrum of each defined autofluorescent compound in the sample and clearly resolves the spatial contribution of each compound.

## Results

### Transmission light microscopy analysis

**Fig. 1:**
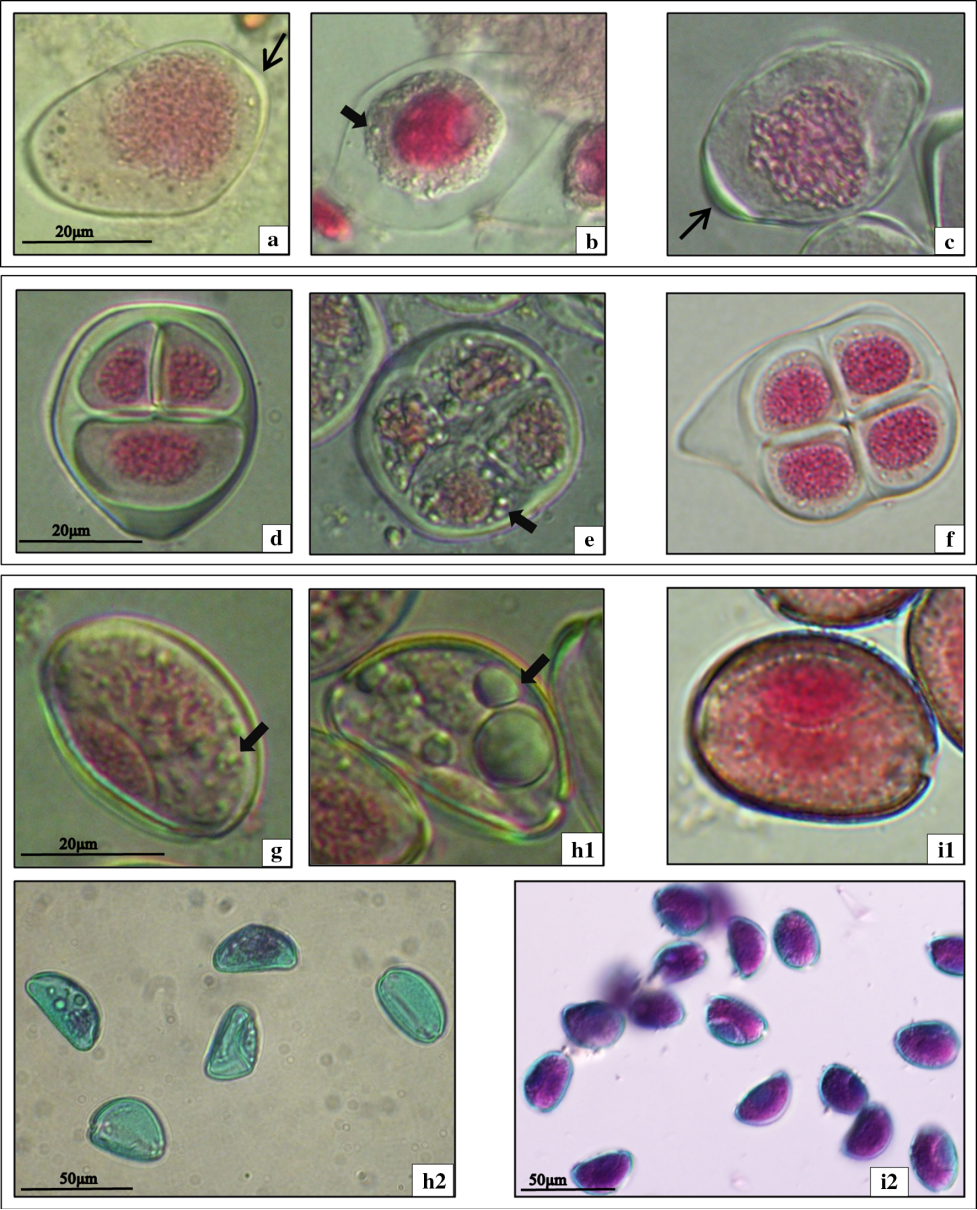
Images from LM with Nomarski contrast. **a**, **b** *A. ampeloprasum* var. *ampeloprasum* (GHG-L): **a** meiocyte at the early prophase I stage (*arrow* callose wall) and **b** degenerating meiocyte (*arrow* vacuole-like vesicles). **c** *A. ampeloprasum* (leek) meiocyte at the early prophase I stage (*arrow* callose wall). **d**, **e** *A. sativum* cv. Harnas: **d** microspore tetrad and **e** degenerating tetrad (*arrow* vacuole-like vesicles). **f** *A. ampeloprasum* microspore tetrad. **g**, **h** *A. sativum* cv. Arkus: **g** bicellular pollen grain (*arrow* vacuole-like vesicles), **h1** degenerating bicellular pollen grain (*arrow* vacuole-like vesicles), and **h2** pollen grains after Alexander test. **i** *A. ampeloprasum*: **i1** bicellular pollen grain and **i2** pollen grains after Alexander test

The analysis of microsporogenesis in the investigated species was performed using a light microscope (LM) with Nomarski contrast. Meiotically dividing cells of *A. sativum* cv. Harnas and Arkus as well as *A. ampeloprasum* var. *ampeloprasum* GHG-L, which are sterile species, were observed with LM. Meiocytes of *A. ampeloprasum* (leek), a fertile species reproducing sexually and producing many seeds, were used as a comparative material. The analysis of the early prophase meiocytes in GHG-L showed the presence of meiocytes resembling cells at the leptotene stage (Fig. 1a). In these cells, the nuclear chromatin did not exhibit a normal degree of condensation of chromatin threads, in contrast to the control cells of the fertile leek (Fig. 1c). In such young microsporocytes, the onset of callose deposition on the outer wall was observed in both the GHG-L and leek (Fig. 1a, c—arrows). The GHG-L meiocytes did not enter the pachytene stage, i.e., the successive phase of meiotic prophase I. These cells exhibited a characteristic, strongly shrunk cytoplasm with numerous vacuole-like vesicles (Fig. 1b, arrow) and a shrunken, homogeneous nucleus without distinct chromosomes (Fig. 1b). In GHG-L, the microsporogenesis process terminated at this stage.

Observation of the *A. sativum* cv. Harnas meiocytes revealed that microsporogenesis proceeded normally until the formation of microspore tetrads (Fig. 1d). The microspore tetrads in cv. Harnas observed by the LM did not exhibit cytological differences from the cells of the fertile leek at the same stage (Fig. 1f). Although there was no release of the tetrad into single microspores, microspore degeneration within the common callose wall was noted. The cytoplasm of such cells had numerous vacuole-like vesicles (Fig. 1e, arrow) and a degenerating nucleus (Fig. 1e). In the Harnas cultivar, there was no gametogenesis stage. By contrast, microsporogenesis in the *A. sativum* cv. Arkus proceeded normally with the typical release of tetrads into single microspores and formation of a bicellular pollen grain. However, such cells did not have a normally formed sporoderm, and their cytoplasm exhibited many small vacuole-like vesicles (Fig. 1g, arrow). The LM image of these cells differed considerably from the image of the leek cells at the same stage (Fig. 1i1). In the *A. sativum* cv. Arkus, the cytoplasm of the bicellular pollen grains underwent degeneration, which was manifested by the presence of numerous large vacuole-like vesicles (Fig. 1h1, arrow). These cells had degenerated vegetative and generative nuclei, as evidenced by the acetocarmine staining (Fig. 1h1). The gametogenesis in cv. Arkus ended at the stage of bicellular pollen grain formation. To determine the viability of the pollen grains in the garlic cv. Arkus and leek, the Alexander test was employed. In the viable *A. ampeloprasum* pollen grains, the protoplast stained purple and the cell wall stained green (Fig. 1i2). Dead pollen grains of the *A. sativum* cv. Arkus stained only green (Fig. 1h2). The Alexander test confirmed that gametogenesis was blocked in Arkus at the stage of the bicellular pollen grain (100 % of non-viable cells), whereas there were 85–90 % of viable pollen grains in the leek.

**Fig. 2:**
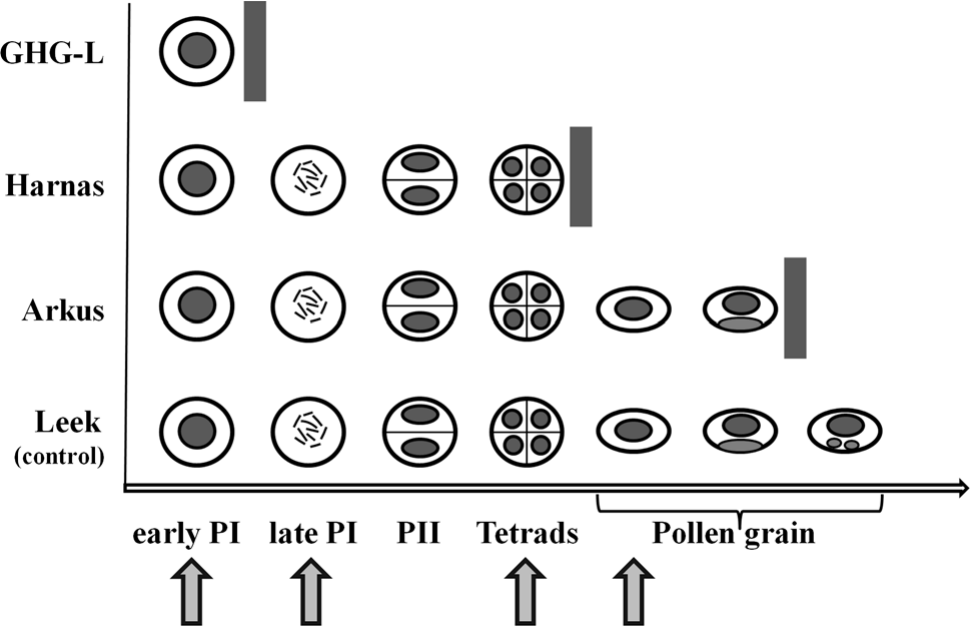
Inhibition at different stages of the male gametophyte development (*grey bar*): *A. ampeloprasum* var. *ampeloprasum* (GHG-L)—early prophase I, *A. sativum* cv. Harnas—microspore tetrad, and *A. sativum* cv. Arkus—bicellular pollen grain. Normal gametogenesis—*A. ampeloprasum* (leek). *Arrows* denote microsporogenesis stages analysed with CLM

These observations indicated that the inhibition of microsporogenesis in the analysed sterile species occurs at different stages of male gametophyte development, i.e., at the beginning of prophase I in GHG-L, at the microspore tetrad stage in Harnas, and at the bicellular pollen grain stage in Arkus (Fig. 2).

### Autofluorescence analysis

To gain deeper insight into the physiology and biochemistry of meiotically dividing cells, the meiocyte callose wall, the pollen grain sporoderm, and the tapetum were individually analysed at the various stages of microsporogenesis. To overcome the limitation of the traditional staining approach, we used autofluorescence-spectral imaging (ASI), since various plant metabolites have fluorescence properties that can be used for cell imaging, based on their biochemical composition. The investigations were conducted on GHG-L and *A. sativum* cv. Harnas and Arkus cells, whose LM images were not cytologically different from the control leek cells. However, the development of the analysed cells at the later stages differed from that noted in the leek cells, which suggested that the disturbances in the microspore development, which were impossible to image with LM, occurred at the earlier stages.

Consequently, we turned our attention to a method that allowed us to have deeper insight into the metabolic changes at the biochemical level, autofluorescence-spectral imaging. ASI can be considered as a very sensitive biosensor for cellular-structural composition biomonitoring, given the correlation between the fluorescence emission spectrum of a particular structure and the chemical composition of that structure (Roshchina 2003). Thus, differences in autofluorescence emission spectra of male gametophytes at various developmental stages may reflect alterations in the chemical composition due to the degradation of one metabolite or accumulation of other components. We applied a qualitative and quantitative approach to study the autofluorescence of meiocytes of all the examined species, using a spectral imaging mode. The samples were excited with UV light (405 nm laser), and the images obtained represented the complete spectral distribution of the fluorescence signals for every point of the image. An autofluorescence software coding function was applied and coding consisted in giving a wavelength-dependent colour to each pixel with intensity proportional to the pixel fluorescence intensity. Using the ASI approach, autofluorescence-spectral-picture encoding clearly demonstrated the cell structure previously described by LM (Fig. 1a–i), but with the addition of a new level of information inaccessible to LM. For all species, the microsporogenesis stages in the analysed cells were identified with transmission light microscopy, using the DIC contrast-enhancing technique, and the identified cells were subsequently examined with the spectral imaging approach.

**Fig. 3:**
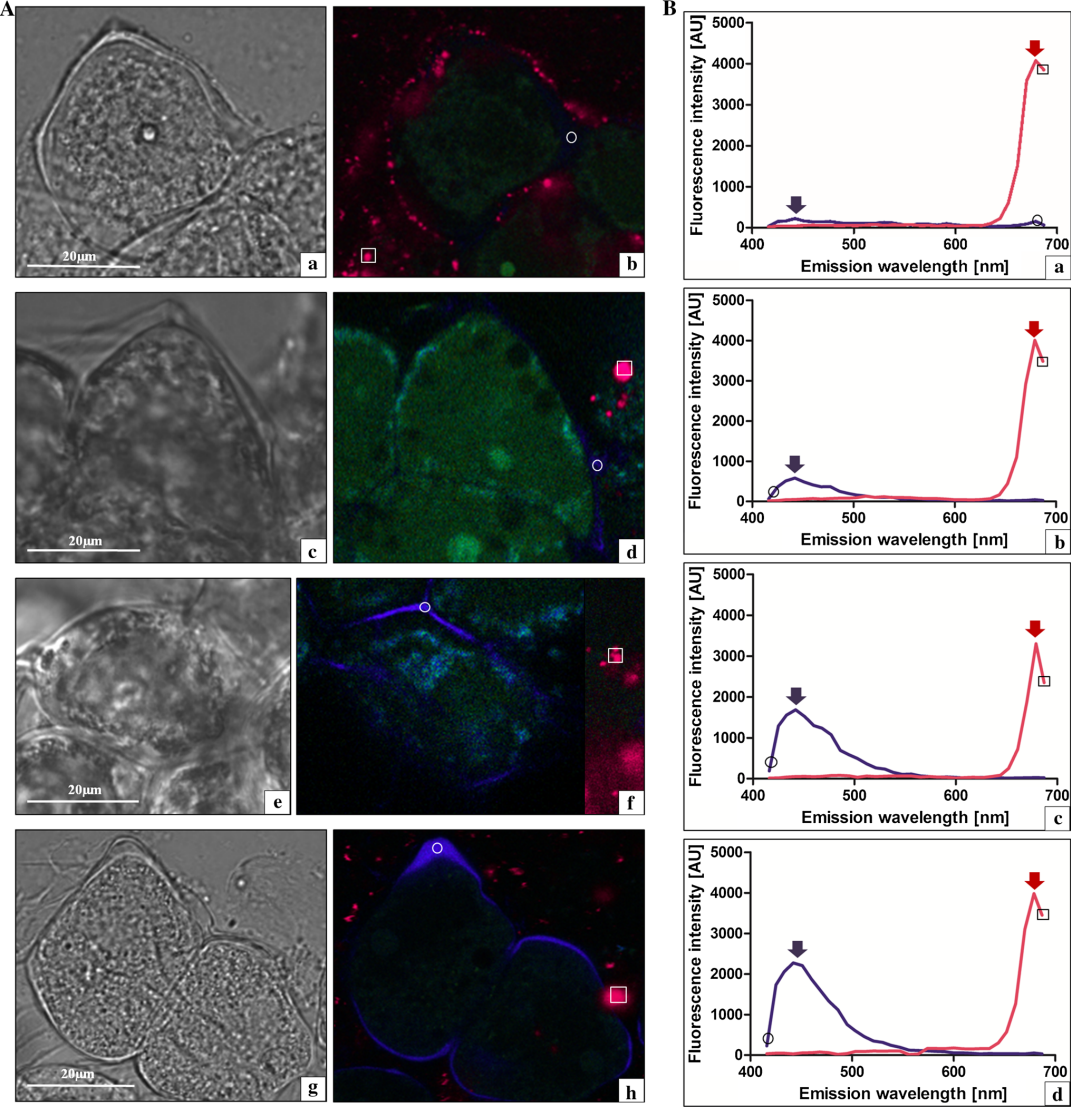
Early prophase I meiocytes and a tapetum fragment in laser scanning confocal microscopy. **A a**, **b** *A. ampeloprasum* var. *ampeloprasum* (GHG-L): **a** DIC and **b** *colour* coded autofluorescence spectral image (ASI), *circle* callose wall, and *square* tapetum. **c**, **d** *A. sativum* cv. Harnas: **c** DIC and **d** *colour* coded ASI (*circle* callose wall and *square* tapetum). **e**, **f** *A. sativum* cv. Arkus: **e** DIC and **f** *colour* coded ASI (*circle* callose wall and *square* tapetum). **g**, **h** *A. ampeloprasum* (control): **g** DIC and **h** *colour* coded ASI (*circle* callose wall and *square* tapetum). **B** Extracted spectra from the *colour* coded autofluorescence spectral images: **a** *A. ampeloprasum* var. *ampeloprasum* (GHG-L); **b** *A. sativum* cv. Harnas; **c** *A. sativum* cv. Arkus; and **d** *A. ampeloprasum* (control). Callose wall *circle* and tapetum *square*

The early prophase I meiocytes of GHG-L (Fig. 3Aa), *A. sativum* cv. Harnas (Fig. 3Ac), cv. Arkus (Fig. 3Ae), and the control *A. ampeloprasum* meiocytes (Fig. 3j) were subjected to 405 nm excitation light, and fluorescence emission light was collected in the range of 410–690 nm for the whole cell (Fig. 3Ab, d, f, h). Special attention was paid to the callose wall of the meiocytes (indicated by a circle), and the tapetum (shown by a square) and emission spectra were extracted and presented in a quantitative/qualitative mode (Fig. 3Ba—GHG-L; Fig. 3Bb—Harnas; Fig. 3Bc—Arkus; Fig. 3Bd—leek). The control cells (leek) exhibited strong autofluorescence of the callose wall in the violet–blue light range (410–490 nm), with maximal intensity at 440 nm (Fig. 3Bd, violet arrow). The emission peak had the characteristic shape of A curve with an extended shoulder on the descending arm. The analysed early prophase I meiocytes in the sterile species exhibited callose autofluorescence in the same spectral range as that in the leek and had a similarly shaped curve. However, the autofluorescence intensity varied between the species. GHG-L showed the lowest intensity of callose autofluorescence (Fig. 3Ba, violet arrow), the cv. Harnas exhibited intermediate intensity (Fig. 3Bb, violet arrow), and the intensity in the cv. Arkus was similar to that noted for the leek (Fig. 3Bc, violet arrow). During the analysis of the spectrum of the autofluorescence of the nutritive tissue (that is, the tapetum), fluorescence emission in the red light range of 650–690 nm with a distinct maximal value at 680 nm was noted in all the investigated species (Fig. 3Ba, b, c, d, red arrow). Neither the intensity nor the maximum of red fluorescence emission of the tapetum differed across the analysed species. Taken together, the results indicate that the callose wall formed in the early prophase I meiocytes of all the species has similar biophysical spectral parameters. However, the fluorescence intensity in GHG-L is low, which suggests substantial changes in the callose composition in comparison with the other species. In turn, the biophysical parameters of the tapetum show that this nutritive tissue in the investigated sterile species did not exhibit qualitative and quantitative differences from the tapetum parameters in the leek, in which the microsporogenesis proceeded normally.

**Fig. 4:**
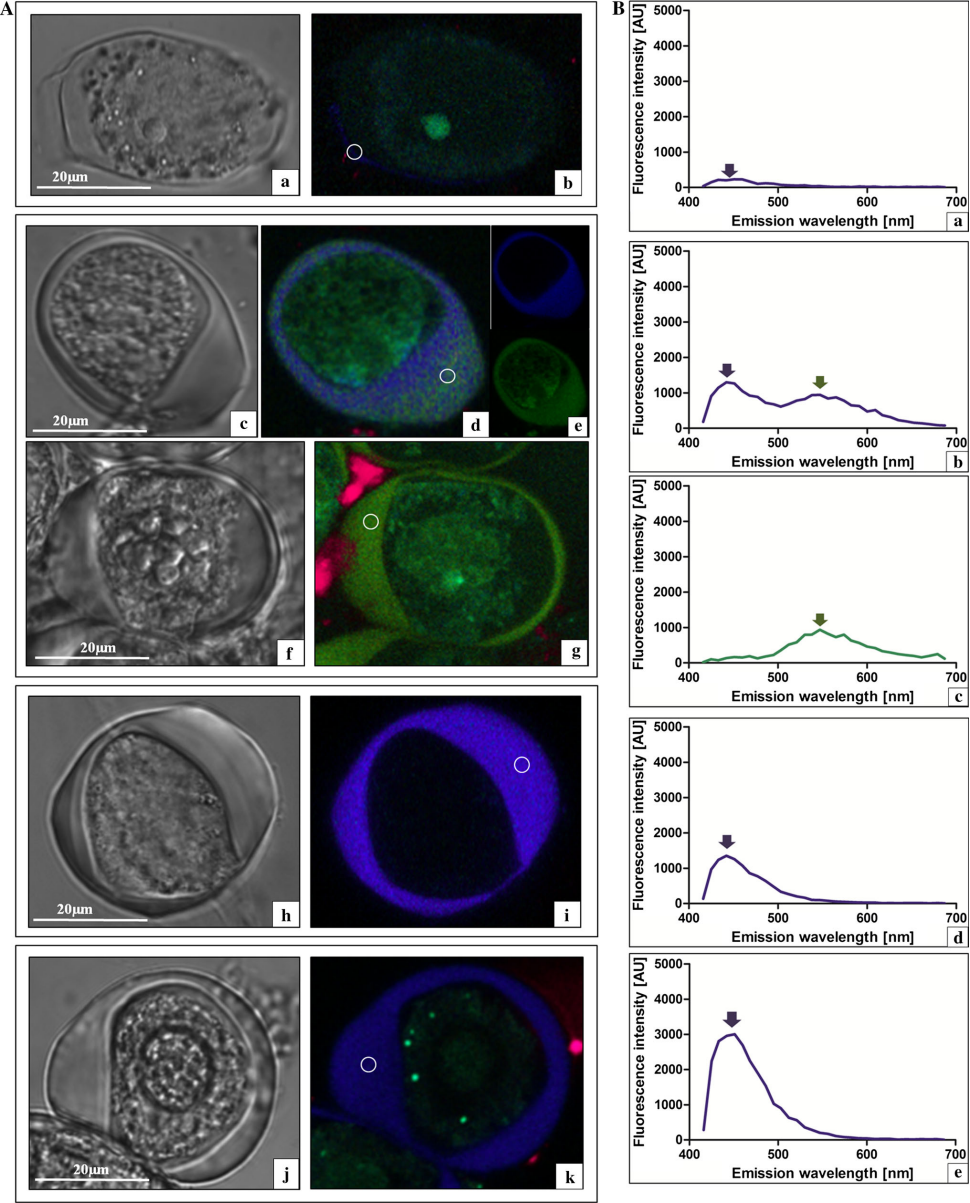
Late-prophase I meiocytes in laser scanning confocal microscopy. **A a**, **b** *A. ampeloprasum* var. *ampeloprasum* (GHG-L): **a** DIC and **b** *colour* coded autofluorescence spectral image (ASI) and *circle* callose wall. **c**–**e** *A. sativum* cv. Harnas: **c** DIC, **d** *colour* coded ASI (*circle* callose wall), and **e** spectral unmixing of the callose wall; **f**, **g** meiocytes at a later prophase I stage (diakinesis): **f** DIC and **g** *colour* coded ASI (*circle* callose wall). **h**, **i** *A. sativum* cv. Arkus: **h** DIC and **i** *colour* coded ASI (*circle* callose wall). **j**, **k** *A. ampeloprasum* (control): **j** DIC and **k** *colour* coded ASI (*circle* callose wall). **B** Extracted spectra from the *colour* coded autofluorescence spectral images: **a** *A. ampeloprasum* var. *ampeloprasum* (GHG-L); **b**, **c** *A. sativum* cv. Harnas: **b** prophase I and **c** later prophase I; **d** *A. sativum* cv. Arkus; and **e** *A. ampeloprasum* (control). Callose wall *circle* and tapetum *square*

Subsequently, the late-prophase I meiocytes of all the investigated species were analysed. The callose wall in the GHG-L cells, which clearly exhibited cytoplasm degeneration at this stage (Fig. 4Aa), was characterised by a very low intensity of callose wall autofluorescence in the violet–blue light range (Fig. 4Ab, Ba). In turn, the late-prophase *A. sativum* cv. Harnas cells (Fig. 4Ac) had a complex spectrum of callose autofluorescence (Fig. 4Ad). Intriguing data were provided by applying the very powerful method called spectral unmixing. This application, using computer algorithms, provides an opportunity to distinguish individual spectral signals recorded from a single pixel containing a number of fluorophores into spatially separated intensity signals of emanating from each of them. The linear unmixing method showed that the callose autofluorescence spectrum in the cv. Harnas had two clear components (Fig. 4Ae). The first spectrum in the violet–blue light range had an emission maximum at 450 nm, and the second spectrum in the green–yellow light range had a maximum at a wavelength of 550 nm (Fig. 4Bb, arrows). Interestingly, the callose wall in the cv. Harnas meiocytes in the late-prophase I—diakinesis (Fig. 4Af) exhibited complete autofluorescence decay in the violet–blue light range and a dominance of the green–yellow light spectrum (Fig. 4Ag, Bc, green arrow) with a maximum at 550 nm. Its fluorescence intensity and spectrum maximum were comparable to that of callose autofluorescence in this range in the earlier prophase I of cv. Harnas meiocytes. In the next step, the prophase meiocytes of the *A. sativum* cv. Arkus (Fig. 4Ah) were analysed. The spectral analysis of the autofluorescence of the callose wall in these cells showed a characteristic spectrum in the violet–blue light range (410–500 nm) with a very high intensity at a maximum of 450 nm (Fig. 4Ai, 4Bd, violet arrow). At the same stage of microsporogenesis in leek (Fig. 4Aj), a similar spectral range of callose wall autofluorescence was noted, although it was substantially less intense (Fig. 4Ak, 4Be, violet arrow). These analyses showed that, already at the prophase I stage, there were substantial qualitative and quantitative differences in the callose wall between the sterile and fertile species. No significant differences in the autofluorescence of the nutritive tissue (the tapetum) were noted (data not shown).

**Fig. 5:**
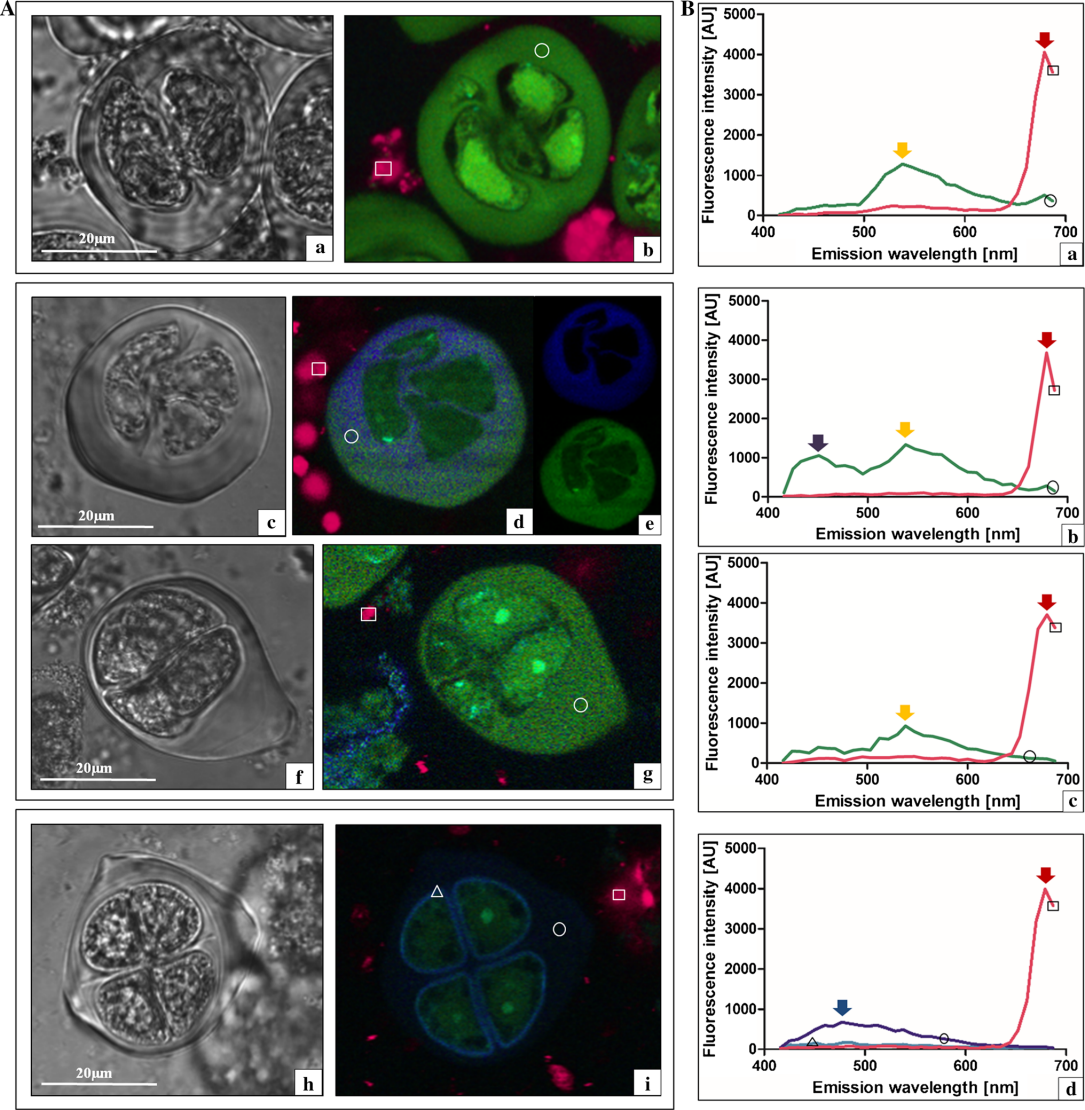
Microspore tetrads and a tapetum fragment in laser scanning confocal microscopy. **A a**, **b** *A. sativum* cv. Harnas: **a** DIC and **b** *colour* coded autofluorescence spectral image ASI, *circle* callose wall, and *square* tapetum. **c**, **d** *A. sativum* cv. Arkus: **c**–**e** young tetrad: **c** DIC, **e** *colour* coded ASI (*circle* callose wall and *square* tapetum), and **f** spectral unmixing of the callose wall; **f**, **g** older tetrad: **f** DIC and **g** *colour* coded ASI (*circle* callose wall and *square* tapetum). **h**, **i** *A. ampeloprasum* (control): **h** DIC and **i** *colour* coded ASI (*circle* callose wall, *triangle* sporoderm, and *square* tapetum). **B** Extracted spectra from the *colour* coded autofluorescence spectral images: **a** *A. ampeloprasum* var. *ampeloprasum* (GHG-L); **b** *A. sativum* cv. Harnas; **c** *A. sativum* cv. Arkus; and **d** *A. ampeloprasum* (control). Callose wall *circle* and tapetum *square*

The next analysis involved the observation of the microspore tetrads in the *A. sativum* cv. Harnas (Fig. 5Aa), cv. Arkus (Fig. 5Ab, f), and leek cells at the same developmental stage (Fig. 5Ah). Once again, it should be underlined that the DIC imaging did not reveal cytological differences between these cells, and that their callose walls exhibited no morphological differences. In contrast, the autofluorescence analysis of the same cells showed essential differences in the callose walls of cv. Harnas, cv. Arkus, and leek (Fig. 5Ab, d, g, i, circle). The spectral analysis of the callose wall surrounding the microspore tetrad showed two clear structures in the control leek sample that emits fluorescence in the violet–blue part of the spectrum. These include the callose wall (emission in the range of 410–600 nm, with a maximum at 480 nm—Fig. 5Bd, blue arrow) and the sporoderm with considerably less intense fluorescence in the same spectral range (Fig. 5Bd, triangle). Contrary to the leek, the callose wall of the cv. Harnas tetrad exhibited predominantly green–yellow autofluorescence in the range of 500–600 nm (with a maximum at 540 nm—yellow arrow), with almost no emission in the violet–blue light range (Fig. 5Ab, Ba). Interestingly, the callose wall surrounding the young Arkus tetrads (Fig. 5Ac) exhibited a complex autofluorescence spectrum (Fig. 5Ad). The linear unmixing method showed that the spectrum had two components (Fig. 5Ae). The first spectrum in the violet–blue light range had an emission maximum at 450 nm, and the spectrum in the green–yellow light range exhibited a maximum at a wavelength of 550 nm (Fig. 5Bb, arrows). The older tetrads of the cv. Arkus (Fig. 5Af) were surrounded by a callose wall, which exhibited autofluorescence decay in the violet–blue light range, whereas the spectrum in the green–yellow light range with a maximum of 550 nm was predominant (Fig. 5Ag, Bc, yellow arrow). Based on these results, it can be concluded that the callose walls in the *A. sativum* cv. Harnas and cv. Arkus at the microspore tetrad stage had a completely different chemical structure than that of the callose wall in the leek. Furthermore, the spectral analysis revealed the absence of the sporoderm, which began to form at this stage in the fertile leek. In contrast, the analysis of the autofluorescence of the nutritive tissue surrounding the cells at this stage did not show substantial quantitative and qualitative differences. Fluorescence emission of the tapetum in the range of 650–690 nm with a very distinct maximum at 680 nm was noted in the all analysed species (Fig. 5Ba, b, c, d, red arrow).

**Fig. 6:**
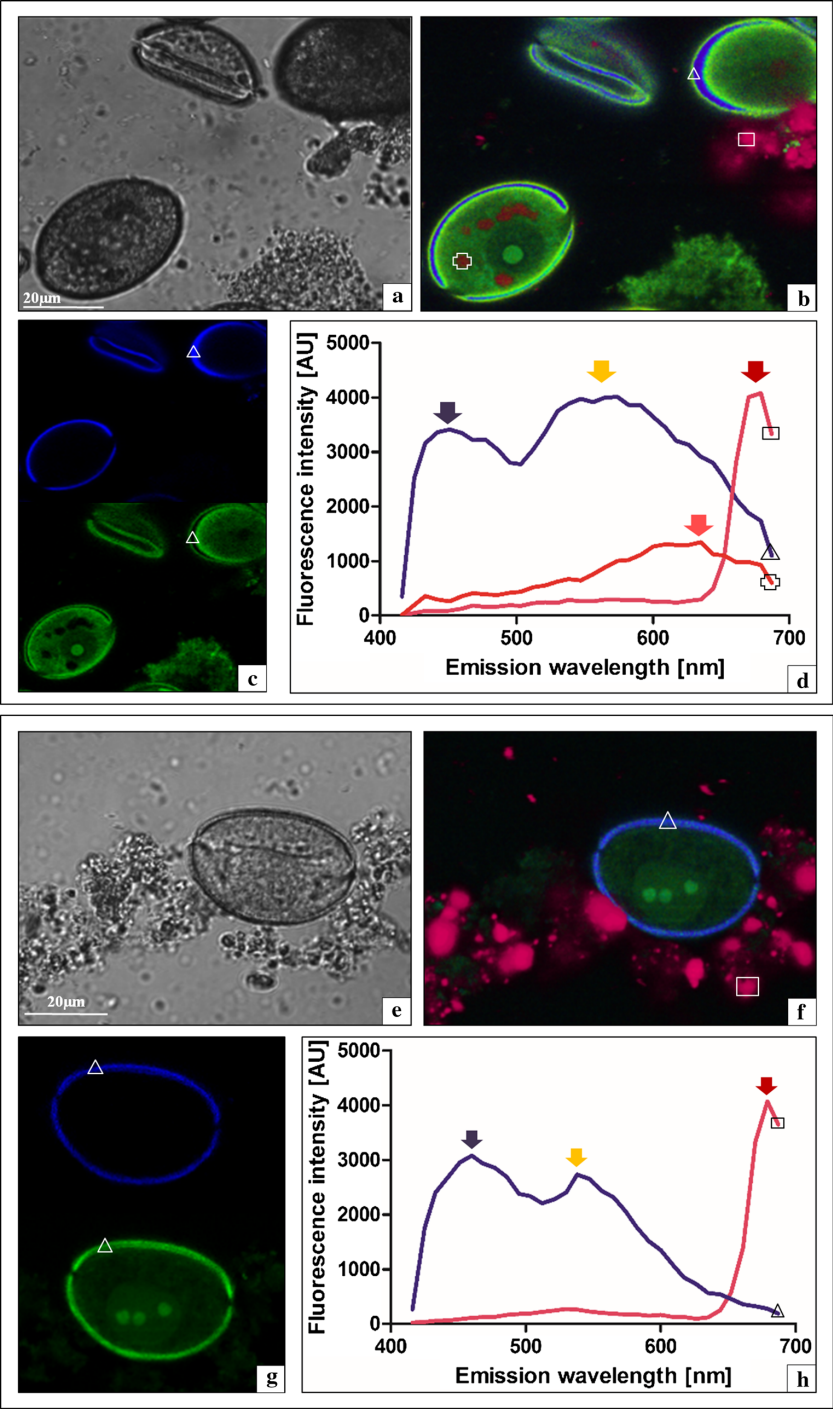
Mononuclear pollen grain and a tapetum fragment in laser scanning confocal microscopy. **a**–**d** *A. sativum* cv. Arkus: **a** DIC, **b** *colour* coded autofluorescence spectral image (ASI), *triangle* sporoderm, *square* tapetum, and *cross* vacuole-like vesicles, **c** spectral unmixing (sporoderm *triangle*), and **d** extracted spectra from the sporoderm (*triangle*), tapetum (*square*), and vacuole-like vesicle (*cross*). **e**–**h** *A. ampeloprasum*: **e** DIC, **f** *colour* coded ASI (*triangle* sporoderm and *square* tapetum), **g** spectral unmixing (sporoderm *triangle*), and **h** extracted spectra from the sporoderm (*triangle*) and tapetum (*square*)

In this study, we also analysed the mononuclear pollen grains of the sterile *A. sativum* cv. Arkus and the cells of the fertile leek at the complementary stage. As shown above, the DIC imaging did not reveal morphological–cytological differences between these cells (Fig. 6a, e). Once again, the autofluorescence-spectral analysis, in contrast to DIC, displayed significant differences in the sporoderm of cv. Arkus, compared to the control leek sample, as shown by the colour coded autofluorescence-spectral image (Fig. 6b, f). As observed using the spectral unmixing method, the sporoderm in the Arkus cultivar and the leek was characterised by complex autofluorescence spectra (Fig. 6c, g). The autofluorescence spectrum of the sporoderm in the cv. Arkus exhibited a broad emission range covering the range in violet–blue and green–yellow light, with two main characteristic emission maxima at 450 and 550 nm (Fig. 6d, violet and yellow arrows). The sporoderm in the mononuclear pollen grains of the leek exhibits ED autofluorescence that covers a broad emission range with similar maxima at 460 and 540 nm, but with significantly different curve paths. This indicates that the ratio between the two main peaks of emission spectra significantly differs from the ratio of cv. Arkus. Notably, the contribution of the second peak at 540 nm to the whole spectrum in the case of the leek is weaker, which suggests that green–yellow-light-emitting compounds are in the minority in the case of the leek, and that violet–blue-light compounds dominate in the sporoderm (Fig. 6h, violet arrow). This analysis shows that the sporoderm of cv. Arkus has a different biochemical composition in terms of amounts of autofluorescent substances, in comparison with the leek. Interestingly, vacuolar-like structures were detected in the cv. Arkus gametophyte using the autofluorescence approach, exhibiting a maximum at 630 nm (pink arrow) clearly separated from the emission spectra of the sporoderm and tapetum (Fig. 6b, d, cross); no such structures were present in the control leek samples. In the case of the tapetum, the recorded spectra exhibited a similar profile in the range of 650–690 nm at a distinct maximum at 680 nm in both species (Fig. 5d, h, red arrows).

## Discussion

Given all the current problems associated with the depletion of resources and increasing demands for efficient food production, finding a solution to those problems related to plant reproduction should be given priority (Scott et al. 2004). One of these issues is plant sterility, which is determined by a number of factors, including morphological, genetic, and biochemical impacts, which often have an anthropogenic background. For example, many developmental and structural anomalies with yet unknown causes can be observed in the ontogenetic development of male-sterile garlic, which is not only an industrially very important plant, but which is also an interesting species in the context of evolution in the human environment (Etoh et al. 1988; Simon and Jenderek 2003).

The investigations presented in this paper show a remarkable diversity of male sterility not only within the genus *Allium* (*A. sativum*—*A. ampeloprasum* var. *ampeloprasum*), but also between cultivars of one species (*A. sativum* cv. Harnas—cv. Arkus). Using the classification proposed by Shemesh Mayer et al. (2013) and based on our observations of the analysed species, we can assign them to the first three types of sterility. *A. ampeloprasum* var. *ampeloprasum* (GHG-L) represents the complete sterile type 1, as disturbances are noted both in the male line, at a very early stage of microsporogenesis, and in the female line (female line—unpublished observations). *A. sativum* cv. Harnas, in which a microsporogenesis inhibition was observed at the stage of microspore tetrads, can be classified as male-sterile type 2. In turn, *A. sativum* cv. Arkus, producing sterile pollen grains, belongs to male-sterile type 3. Such diverse manifestations of sterility suggest high complexity of the microsporogenesis and gametogenesis processes and indicate that pollen abortion in plants from the genus *Allium* can occur at any stage of male gametophyte development.

Male-sterile plants often exhibit microsporogenesis disturbances involving the reduction or disorganisation of the cytoplasm, absence or substantial reduction of the callose wall, and abnormalities in the development of the exine and intine in the pollen grain (Sawhney and Shukla 1994). In the sterile species analysed in this study, the cytological picture of the disturbances in the microsporogenesis process was manifested by remarkable accumulation of vacuole-like vesicles and substantial distortion of the cell nucleus. Similar irregularities in the ultrastructure of the early prophase meiocytes during microsporogenesis have been described in the male-sterile *Petunia hybrida* (Bino 1985) and in *Arabidopsis thaliana* mutants (Peirson et al. 1996).

The abnormalities in the structure of the meiocytes in GHG-L as well as *A. sativum* cv. Harnas and Arkus observed under the transmission light microscope reflect the final stage of the death of the male gametophyte. However, the changes in the course of microsporogenesis must have begun at the earlier stages of the process, although they were not discernible in LM. To identify the early metabolic changes preceding the evident (visible in LM) death of the male gametophyte, we performed autofluorescence-spectral-imaging analysis, which provides an additional cognitive level, supplementing the traditional anatomical and cytological analyses with biochemical insight.

Autofluorescence spectral imaging is a non-invasive optical technique that does not require tedious staining or other pre-treatment techniques. It is a very effective approach allowing differentiation of naturally occurring fluorophores in plant cells. The optical properties of plant cells make them very suitable for studying metabolic fluctuations using the autofluorescence emission properties of many plant components. For instance, the main and very well-characterised fluorescent component is chlorophyll, which has been studied extensively using autofluorescence as a means of non-invasive evaluation of photosynthesis and the physiological status of plants and/or of the effect of environmental stresses on plants (García-Plazaola et al. 2015). However, chlorophyll is only one example among the many fluorescent compounds that have been found in plants. In particular, some very interesting fluorescent compounds include phenolic compounds (e.g., anthocyanins, flavonoids, tannins, and cinnamic acids), alkaloids (e.g., betalains), terpenoids, and porphyrins (e.g., chlorophyll as the best characterised compound, chlorophyll catabolites, and heme-containing proteins). All these metabolites possess specific physical properties and, upon excitation with UV light, emit specific fluorescent signals in the whole range of light spectra, covering violet (400–455 nm), blue (460–490 nm), green–yellow (500–590 nm), and red (600–750 nm) light. Importantly, every compound from the specific group of fluorescent metabolites generates specific emission spectra and can be partially recognised based on these properties, which may provide a valuable information about plant physiological conditions, plant diseases, and many abiotic stresses, such as nutrient deficiencies (Usha and Singh 2013). Many of the autofluorescent compounds are metabolites produced and/or accumulated in particular tissues or specialised cells. In many cases, they are involved in biotic or abiotic interactions of plants with their environment (Bellow et al. 2012). Thus, the fluorometric detection of these compounds can be used for the qualitative study of their role and dynamics in plant cells, as has been shown for coffee tree leaves, which were subjected to the spectral analysis focused on the histochemical differentiation of those leaves (Conéjéro et al. 2014).

Given the great analytical potential of autofluorescence spectral imaging, we used this method for the analysis of the changes in the microsporogenesis process with a focus on biochemical fluctuations in the meiocyte callose wall at all the stages of meiosis. It is well known that the callose wall formed during microsporogenesis plays a very important role in the cytokinesis and formation of the primary septum, in the early deposition of sporopollenin around the microspores, and in the subsequent formation of a specific post-meiotic wall (Dong et al. 2005). At the initial stages of normal microsporogenesis, the amount of callose increases, while at the final meiotic stage, callose is gradually degraded by the callase enzyme. Currently, it is agreed that callose may have fundamental importance in the production of functional pollen grains (Fei and Sawhney 1999; Enns et al. 2005; Teng et al. 2005). Therefore, based on the ASI analysis of the first microsporogenesis stage (early prophase I), we noted that the autofluorescence spectra of the callose wall observed in all the investigated species were characterised by the same spectral distribution (violet light range) and maximum, but differed in the autofluorescence intensity. Compared with the control (leek), greater differences were observed when the microsporogenesis blockade occurred earlier. Particularly, striking observations were made for GHG-L, in which the inhibition of the development of meiocytes was noted already in prophase I and the callose wall emitted very weak autofluorescence in the violet light range. Interestingly, although the microsporogenesis inhibition in *A. sativum* cv. Harnas occurred only at the microspore tetrad stage, the spectrum exhibited considerably lower intensity than in the fertile control. This phenomenon may indicate disturbances in the deposition of autofluorescent compounds present in the callose wall already at the early microsporogenesis stage, and probably, such disturbances lead to lack of disintegration of the wall at the microspore tetrad stage in cv. Harnas and, hence, no gametogenesis. The spectrum with a maximum at 450 nm obtained at this stage of the callose wall development probably represents the group of hydroxycinnamic acids (most likely—ferulic acid) (Knogge and Weissenboèck 1986). As reported by Miranda et al. (1981), the level of ferulic acid in the cell wall is correlated with the development of the plant cell and the decline in its level is associated with plant cell ageing. The phenomenon of fluorescence fluctuations in the violet light range is confirmed by the subsequent observations of late-prophase I, where the dying GHG-L microspore exhibited almost complete violet light autofluorescence decay. In turn, in *A. sativum* cv. Arkus, in which microsporogenesis was blocked at the latest stage, both the range and the intensity of autofluorescence were comparable to those in the fertile control.

Extremely interesting results were provided by the spectral analysis of the callose wall in *A. sativum* cv. Harnas at the late-prophase I stage, which revealed subtle changes, invisible in LM, in the callose wall composition reflected in a complex spectrum in the violet–blue light range and additional autofluorescence in the green–yellow light range. At the next stage of the late-prophase I in cv. Harnas, there was complete autofluorescence decay in the violet–blue light range and dominance of the green–yellow light spectrum. These results were completely different from the fertile control, which implied that there were no hydroxycinnamic acids (ferulic acid or *p*-coumaric acid) in the callose wall of cv. Harnas at this microsporogenesis stage. In turn, the dominant spectrum in the green–yellow light range may suggest the presence of many phenolic compounds (chlorogenic acid), flavins (riboflavin, flavoproteins), and terpenoids (carotenes and xanthophylls) (García-Plazaola et al. 2015), which were not observed in either *A. sativum* cv. Arkus or the leek. At that stage, the callose wall surrounding the *A. sativum* cv. Harnas meiocytes exhibited only a green–yellow light spectrum, until the formation of microspore tetrads. At this stage, there was no gradual degradation of the callose envelope and release of single microspores, which gradually died instead.

A similar phenomenon involving the appearance of the green–yellow light spectrum of the callose wall was observed in *A. sativum* cv. Arkus in the microspore tetrad stage. In cv. Arkus, the callose wall spectrum was at first composed of two components as well; next, the violet–blue light autofluorescence intensity in the mature tetrad declined and the green–yellow light spectrum dominated. Changes in the cv. Arkus callose wall composition resemble the abnormalities observed in the late-prophase cv. Harnas; in contrast to cv. Harnas; however, the spectrum in the violet–blue light range did not disappear completely. It can be assumed that the presence of even small amounts of compounds from the group of hydroxycinnamic acids in the callose wall of cv. Arkus contributes to the disintegration of the tetrad into single microspores. This is supported by the observations of the fertile control, in which the callose wall exhibits autofluorescence only in the violet–blue light range throughout the microsporogenesis process; hence, it can be concluded that the presence of hydroxycinnamic acids in the callose wall seems to be indispensable for normal development of the male gametophyte. Notably, in the tetrad stage of the fertile leek, the autofluorescence maximum is shifted towards the blue–green light range (fluorescence maximum at 470 nm), which indicates the presence of phenolic compounds, e.g., coumarins and esculetin, which have a fluorescence maximum at 475 nm, or tannins (500 nm). The spectrum may also be generated by alkaloid compounds, such as betalaines and quinoline alkaloids (510–530 nm) and terpenoids, e.g., carotenoids (500–525 nm). These results suggest that biochemical changes in the callose wall are associated with the normal gradual disintegration of this structure.

In the final stage of the investigations, we analysed the biophysical parameters of the sporoderm of the mononuclear pollen grain in *A. sativum* cv. Arkus and *A. ampeloprasum*. This is an important structure, and many post-meiotic developmental disorders associated with the formation of the sporodermal wall around pollen grains have been described in sterile *Arabidopsis* mutants. An untypical exine layer in the sporoderm of mutants *ms*2 and *dex*1, abnormally deposited sporopollenin in mutant *nef*1, and absence of the intine in the sporoderm of mutant *ms*33 have been described (Ma 2005). Similar findings were reported in sterile *A. sativum* L13, where the sporoderm was devoid of the intine (Winiarczyk 2009). In the species analysed in this study, the pollen grains had a well-developed sporoderm differing in the spectral parameters. It is worth noting that the fertile leek exhibited the dominance of the spectrum in the violet–blue light range with the minor contribution of green–yellow light, in contrast to that in the sterile cv. Arkus, for which the spectrum in the green–yellow light range dominated. These results suggest that the dominance of compounds generating green–yellow fluorescence may contribute to the metabolic disorders and, consequently, the sterility in *A. sativum* cv. Arkus.

## Conclusion

Using various imaging techniques (LM and ASI), we have shown that the inhibition at the different microsporogenesis stages in the analysed sterile species occur at diverse stages of male gametophyte development, and are associated with compositional fluctuation within the meiocyte callose wall. The use of modern techniques for live-cell imaging—ASI—allowed the observation of quantitative/qualitative variations of autofluorescent chemical compounds within the callose wall. Especially, we have shown that compounds with violet–blue autofluorescence, such as hydroxycinnamic acids, may have a pivotal role during the normal development of the male gametophyte in species which belong to the genus *Allium*. The biophysical characterisation of the metabolic disturbances in the callose wall at the various steps of microsporogenesis provides insight into the molecular basis of male sterility in *A. sativum*. Using the ASI method, it was possible, for the first time, to determine precisely the meiosis stage in which normal microsporogenesis is disturbed, which was not visible using LM. In practical terms, these studies can greatly facilitate the selection of garlic clones exhibiting the least deviation from normal microsporogenesis and gametogenesis during the development of the male gametophyte, which corresponds to the global investigations focused on overcoming male sterility in the garlic, and selection of the best cultivars or ecotypes. In addition, our analyses indicate that the ASI technique is a valuable research tool expanding the possibilities of the conventional cytological–embryological studies.

### *Author contribution statement*

DT—conceived the study, performed experiments, interpreted the data, wrote the manuscript; KD—contributed to Figs. 3, 4, 5, and 6; KW—interpreted the data.
